# Phytochemical Screening and Antioxidant Activities of White and Red Wines from Different Varieties and Wine Regions in Romania

**DOI:** 10.3390/antiox14050564

**Published:** 2025-05-08

**Authors:** Ovidiu Tița, Petronela Anca Onache, Elisabeta-Irina Geana, Corina Teodora Ciucure, Dorin Ioan Sumedrea, Alina Florea

**Affiliations:** 1 Department of Agricultural Sciences and Food Engineering, Lucian Blaga University of Sibiu, 550024 Sibiu, Romania; ovidiu.tita@ulbsibiu.ro; 2 National Research & Development Institute for Biotechnology in Horticulture Stefanesti-Arges, Sos. Bucuresti-Pitesti, No. 37, 117715 Pitesti, Romania; dsumedrea@yahoo.com (D.I.S.); alinaflorea964@gmail.com (A.F.); 3 National Research & Development Institute for Cryogenic and Isotopic Technologies-Ramnicu Valcea Romania, Strada Uzinei, No. 4, 240050 Râmnicu Vâlcea, Romania; corina.ciucure@icsi.ro

**Keywords:** polyphenolic compounds, antioxidant activity, phytochemicals, wine

## Abstract

The characteristics of the variety from which the wine is made, the geographical area of production, the year of production, and the technology of winemaking are the parameters with the most influence on the total content of polyphenols, the polyphenolic profile, and the antioxidant activity of wine. For this reason, a polyphenolic screen can help establish the authenticity of wines. For this study, 50 samples of white and red wine from different wine areas were collected. For these samples, a qualitative and quantitative analysis was carried out on the polyphenolic profile. The polyphenolic profiles of the studied wines were determined using UHPLC-ESI-MS/MS (mass spectrometry with tandem ionization with high-performance liquid chromatography). Among the non-flavonoid acids, gallic acids, p-coumarnic, and syringic acids in red wines showed higher concentrations in all samples, while resveratrol was present in concentrations from 0.605 to 12.38 mg/L in red wines, and white wines ranged from 0.07 to 0.35 mg/L. For flavonoids, -catechin (0.187 m/L–130.98 mg/L in red wines and 0.04–4.45 mg/L) and (-)-epi-catechin showed the highest concentrations, reaching up to 29.78 mg/L in red wines.

## 1. Introduction

Grapes are the most cultivated fruit in the world, and therefore, wine can be considered among the most popular food products [1]. This food contains phytochemicals with undeniable properties. A total of 80% of the grapes grown are grapes for wine production [1,2], and they contain various polyphenolic compounds, which are extracted in the wines during winemaking and give wines their undeniable properties [3,4]. Due to the modification of polyphenolic compounds during fermentation, various research fields studying phytochemical composition and antioxidant activity in wines have emerged [5]. The concentration and composition of polyphenolic compounds in wines depend on many factors, such as grape variety [5,6], climate [7,8], soil composition [7,8], grape maturation conditions [8,9,10,11], viticulture techniques [10], and winemaking processes [11,12].

Polyphenolic compounds in wine are bioactive substances, intensively studied [5], not only because of their organoleptic role (contributes to astringency and bitterness) but also because of their property to prevent degenerative processes [5,13], such as certain cancers [14], chronic inflammation and thrombosis [15], cardiovascular disorders and osteoporosis, and diseases associated with oxidative DNA damage, proteins, and lipids [16,17,18]. The antioxidant activity of polyphenolic compounds in wines, especially resveratrol and polyphenols that suppress the appearance of free radicals [5], can help limit these types of damage [13] by stimulating defense systems [13,17]. Flavonoids (flavonol, flavan-3-ol, and anthocyanins) [5] and non-flavonoid compounds (phenolic acids, phenolic alcohols, and stilbens) [19] are strong antioxidants in wine; their chemical structure allows them to act as antioxidants, eliminating and neutralizing free radicals [20]. In white wine, flavonoids account for 20% of all polyphenolic compounds [21]. The browning of white wines [22,23] is responsible for the flavonoid sub-class flavanols, which participate in the chemical oxidation reactions in wines and are responsible for the taste properties of white wines [24]. Among the non-flavonoid class, hydrocynamic acids account for 50% of the total polyphenolic compounds in white wines [22,23] and are responsible for the oxidation of wine [24].

Due to the antioxidant activity of wine and its importance for human health, it has led to multiple research studies and different methods of determination. Research on the antioxidant action of wine, as a food, has led to conflicting results and is often hard to compare [19] due to the methodology used and the absence of a standardized method [2,25]. Many methods have been developed to determine antioxidant activity, including free radical reduction and lipid peroxidation assays [2].

Wine, being a complex food product, during storage and aging, undergoes various transformations and various chemical reactions between wine compounds, including the transformation of polyphenolic compounds. Polyphenolic compounds are considered by some authors a chemical marker for confirming the authenticity of the variety and the geographical origin of grapes and wines [26]. In recent years, anthocyanin profiles have been characterized and used for the classification and differentiation of grape varieties and monovarietal wines [26,27,28,29].

The endorsement of the authenticity of wines from a particular area of production of raw material is of particular importance both for consumers and for producers in general. The more complete and well grounded the data available in this respect, the more accurate and conclusive the results regarding wine authentication. Of great importance is the identification of the representative markers against which the appreciation of the authenticity of wines, and in this case, we have pursued the authentication of wines from recognized wine-growing areas in Romania (Stefanesti, Babadag, Romania, Focsani, Cotnari, Iasi, Blaj, Tarnave, Minis, and Silagiu) depending on the content of polyphenolic compounds, by varieties and years of study. We evaluated the antioxidant activity of these wines, white and red, by studying the antioxidant activity in comparison with the total content of polyphenols, the total content of catechins, and the total content of tannins; in the red wines, we added the total content of anthocyanins and pigments, expressed by the intensity of the dyes and the hues of the wine. A polyphenolic profile for the studied white and red wines was also developed on this occasion.

## 2. Materials and Methods

### 2.1. Wine Samples

Monovarietal wines, 35 white wine samples (Table 1) and 30 red wine samples (Table 2), from 9 famous wine areas in Romania (Babadag, Focsani, Silagiu, Stefanesti, Dragasani, Cotnari, Cotnari, Iasi, Tarnave, and Blaj wine center) were used. The geographical areas (Table 1 and Table 2) where the wine samples were collected are the Stefanesti wine center, South of Romania (Muntenia Hills) (latitude 44°51′ N and longitude 24°57′ E, 250 m altitude), Dragasani vineyard, West of Romania (Oltenia Hills) (latitude 44°39′36.81″N and longitude 24°14′05.86″ E, 201 m altitude), and Babadag Vineyard, South-East Romania (Dobrogea Hills) (latitude 44°53′36″ N and longitude 28°42′43″ E, 250 m altitude), Focsani vineyard, South-East Romania (Vrancea Hills) (latitude 45°42.3′30.4″ N, longitude E-27°08′27.9″, and 142 m altitude), Iasi wine center (latitude 47°12′42.5″, longitude E-27°31′47.1″, and 120 m altitude) and Cotnari vineyard (latitude 47°21′25″ N, longitude E-26°55′10″, and 312,2 m altitude) in Eastern Romania (Moldova Hills), Blaj and Tarnave wine center (latitude 46°10′51.2″ N, longitude E-23°55′40.5″, and 330 m altitude) the center of Romania (Transilvanya Hills), Silagiu vineyard and Minis wine center, (latitude 46°14′51″, longitude E-21°39′52″, and altitude 176 m) in the West of Romania (Banat Hills).

Both, the white and red wines were obtained in the 2019 and 2021 production years. The white wine varieties investigated in this study were Riesling Italian, Fetească Regală, Sauvignon Blanc, Pinot Gris, Muscat Ottonel, Riesling de Rihn, Crâmpoșie Selecționată, Fetească Albă, Tămâioasă Românească, Aligote, Chardonnay, Traminer Roz, Mustoasă de Moderat, and Grasă de Cotnari (Table 1), while the red wine varieties were Burgund Mare, Cabernet Sauvignon, Fetească Neagră, Merlot, Pinot Noir, Negru de Drăgășani, Cabernet Franc, Cadarcă, and Pinot Verdot (Table 2).

### 2.2. Spectrophotometric Analyses

Spectrophotometric determinations (TPCs—total polyphenol compounds, TCCs—total catechin compounds, TTCs—total tannic compounds, TACs—total anthocyanin compounds, AA—antioxidant activity, CI—color intensity, and H—hue) of the wines were performed using a Specord 205 UV/VIS spectrophotometer (Analytic Jena, Konrad-Zuse-Str. 1, Jena/Germany) equipped with 1 cm path length quartz cells.

Total polyphenol compounds (TPC) were determined by Singleton et al.’s method with a Folin–Ciocalteu reagent [30,31], which is the most common and economically analytical method, using gallic acid as a reference standard [32] and measuring the maximum absorbance at 760 nm with the aid of a UV-VIS spectrophotometer. Analytical parameters (color hue and color intensity for red wine) were determined according to the OIV methods [33]. The sample reflection was measured in wavelength ranges of 420, 520, and 620 nanometers. The intensity of the color was calculated by their sum, and the hue was calculated using the ratio between the reflection readings at the wavelengths of 420 and 520. The Folin–Ciocalteu phenol reagent (pure) was obtained from Carl ROTH GmbH Co (Karlsruhe, Germany), and analytical standard gallic acid ≥ 99% was purchased from Carl ROTH GmbH Co (Karlsruhe, Germany).

Total Catechin Compounds (TCCs). The catechin content of wine samples was determined using the method of reaction with vanillin and was described by Amerine and Ough [34]. The vanillin test is specific for proanthocyanidins. The method of determination of catechins is based on the reaction of phloroglucinol with vanillin and produces a reddish color, stable in acid solution. The sample is read at the wavelength of 500 nanometers and is used as standard at the calibration curve (+)-catechin [32]. The analytical standard vanillin ≥ 99% was purchased from Carl ROTH GmbH Co (Karlsruhe, Germany). H_2_SO_4_ 96% was purchased from Chemical Company (Bucharest, Romania).

Total Tannic Compounds (TTCs). The leukoanthocyanid determination method of tannins is based on the property of tannins to transform at high temperatures and in a strongly acidic environment in cyaniding that is red in color [32]. HCl 37% was purchased from Chemical Company (Bucharest, Romania).

Total Anthocyanin Compounds (TACs). The anthocyanin content in wines was determined using the bisulfite bleaching Riberau-Gayon method [34]. Sulfur dioxide additions cause changes in absorbance (520 nm) in the unpolymerized pigments but not in the polymerized pigments [35].

Antioxidant Activity (AA). The wines’ antioxidant activity was determined by the method of Wang et al. [36], as antiradical activity against the stable product DPPH• (2.2–diphenyl-1-picrylhydrazyl) (Sigma Aldrich, Germany). For this purpose, the tested red wine was diluted immediately before the analysis with distilled water. Briefly, 0.1 mL of 0.25 mMol/L freshly prepared DPPH solution was added to 0.1 mL of the sample and homogenized; then, the mixture was incubated at room temperature for 30 min in the dark [32]. The absorbance was measured at 517 nm, and gallic acid was used as a standard on the calibration curve. The radical scavenging assay reagent DPPH, 95% (1,1-diphenyl-2-picryhidrazyl), was purchased from Acros Organics (Slovakia), and Trolox, 97% (6-hydroxy-2,5,7,8-tetramethyl-2-carboxylic acid), was purchased from Alfa Aesar (Thermo Fisher GmbH Kandel, Kandel, Germany).

### 2.3. Phytochemical Profile by UHPLC–ESI/HRMS

The quantitative analysis of polyphenolic profile for individual phenols (phenolic acids, flavonoids, and stilbens) was performed by UHPLC–ESI/HRMS using an UltiMate 3000 UHPLC system (Thermo Fisher Scientific, Bremen, Germany) coupled with a Q ExactiveTM Focus Hybrid Quadrupole—OrbiTrap equipped with HESI). Detailed chromatographic and mass spectrometric conditions were presented in our previous papers [31,37]. The data were processed using the Xcalibur software package (Version 4.1).

Identification of individual polyphenols in wine samples was carried out by correlating the mass spectra and the fragmentation patterns with standard compounds, while the quantification was performed using the standard calibration method.

All chemicals and solvents used in chromatography were obtained from Merck Co. (Darmstadt, Germany), and they had HPLC-MS quality. Analytical standards were purchased from Sigma-Aldrich (Steinheim, Germany).

### 2.4. Statistical Analyses

All the results are expressed as the mean standard deviation (SD), obtained from two independent experiments. Statistical analysis of the results was performed using a Pearson correlation test with a 0.05 significance level. The statistical analysis was carried out, distinct between the different varieties of red and white wine, respectively. All the mathematical and statistical analyses were performed using Microsoft Excel 2010 and XLSTAT add in soft version 15.5.03.3707.

## 3. Results and Discussion

### 3.1. Total Phytochemical Content and Antioxidant Activity

The results from spectrophotometric analyses of active biocompounds and antioxidant activity in white and red wines are shown in Table 3 and Table 4. Table 3 shows the synergy between the total polyphenols, catechin content, and tannin content of white wine varieties and their antioxidant activity. It is noted that the antioxidant activity is directly proportional to the total content of polyphenols. An example is Sauvignon Blanc de Stefanesti since 2021, with the highest antioxidant activity of 78.97 ± 0.693 mgGAE/L and a high content of total polyphenols, 983 ± 0.536 mg GAE/L. The total polyphenol content of wine of the Riesling variety 2019 (294 ± 1.414 mgGAE/L) and from the year 2021 (302 ± 0.364 mgGAE/L) in Stefanesti is somewhat higher than found in the bibliography [37,38,39,40,41], having an average value of 206 mgGAE/L for Riesling 2017; the difference in concentration can also be given by the different production year of the bibliography. The PFT content of Feteasca Regala (397.52 mgGAE/L) in the bibliography [38,39,40,41] is close to that of Dragasani area 2021 (328 ± 1.577 mgGAE/L) and the one in Stefanesti (402.56 ± 1.208 mgGAE/L). The total polyphenol content of the wine of the Feteasca Regala 2019 variety (294 ± 1.414 mgGAE/L) is similar to that found in the bibliography [38,39,40,41], Feteasca Regala 2017 (Stefanesti, 279 mgGAE/L and Vrancea, 296 mgGAE/L). Sauvignon Blanc 2019 (273.292 ± 1.199 mgGAE/L) from Stefanesti is similar to Sauvignon Blanc 2017 from the bibliography [40] (wine from Stefanesti, 241 mgGAE/L). In the Sauvignon Blanc 2019 wine from Focsani (314 ± 0.708 mgGAE/L), the total polyphenol content is a little higher than found in the bibliography [38,39,40,41] for Sauvignon 2017 (252 mgGAE/L). The total polyphenol content of Chardonnay wine 2019 (334.29 ± 0.827 mgGAE/L) and Chardonnay 2021 (397.89 ± 1.351 mgGAE/L) in Babadag is similar to the Chardonnay wine in the bibliography [38,39,40,41] (respectively, 329.89 ± 9.41 mgGAE/L, 339.72 ± 9.41 mgGAE/L, and 396.26 ± 23.41 mgGAE/L).

In Table 3, it is noted that Pinot Gris from Babadag with the 2021 production year has the highest total content of polyphenols, 1349.44 ± 0.17 mg GAE/L, compared to the bibliography [38,39,40,41], where Pinot Gris, production year 2008, in Vrancea has a total polyphenol content of 516.628 mg GAE/L. In the PG21_B sample, even though it has a high polyphenol content, the antioxidant activity is 56.34 ± 0.827 mg GAE/L, which is a high value but not one of the highest (Table 3). The highest values of antioxidant activity are found in SB21_St varieties (78.97 ± 0.693 mg GAE/L) and MO21_St (73.88 ± 0.778 mg GAE/L). The lowest total content of polyphenols (240.22 ± 0.364 mgGAE/L) is found in the Aligote 2019 wine variety from the Babadag vineyard, having a fairly high antioxidant activity (65.705 ± 0.877 mgGAE/L); this shows that these interconnections between total polyphenols and the activity of the antioxidants depend on the wine variety and the geographical area from which the wine is produced.

**Figure 1:**
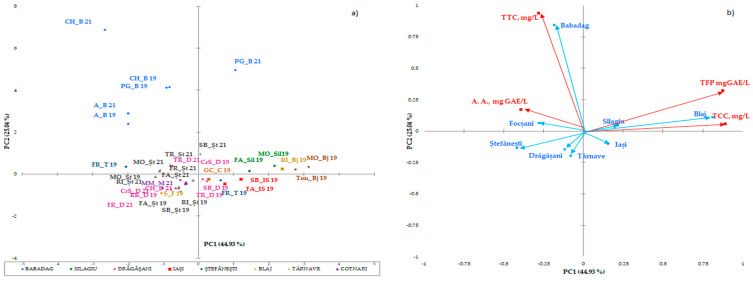
Biplot of the main components, 1 and 2, for the mean scores of the phenolic composition of the wine studied in geographical areas (**a**) and white wine varieties (**b**). The codes are as follows: (TPF) total polyphenolic content, (TCCs) total catechin compounds, (TTCs) total tannic compounds, and (A.A.) antioxidant activity.

**Figure 2:**
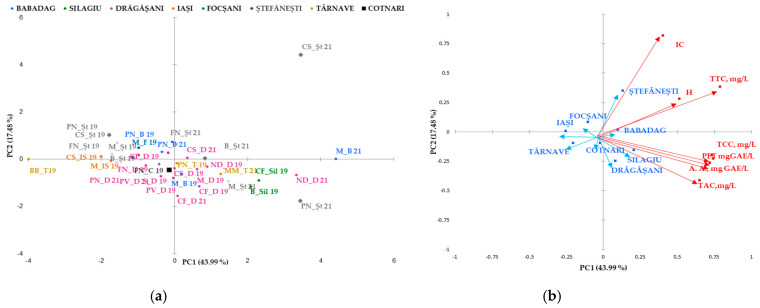
Biplot of the main components, 1 and 2, for the mean phenolic composition scores studied in geographical areas (**a**) and for red wine varieties (**b**). The codes are as follows (CI) color intensity, (H) color hue, (TPF) total phenolic content, (TCC) total catechins compounds, (TTC) total tannic compounds, (TAC) total anthocyanin compounds, and (A.A.) antioxidant activity.

The statistical multivariate analysis was carried out by separating the white wines (Figure 1a,b) from the red wines (Figure 2), due to the major difference between the phytocomponents in the red wines, compared to the white wines. PCA has been applied separately to the two collections of phytochemicals. The first two axes (shown in the diagram) explain 69,97% of the total variation in the phytochemical composition. The maps defined by PC1 and PC2 for PCA on polyphenolic data on the white wine varieties analyzed are shown in Figure 1a and Figure 1b, respectively. The two main components considered 43.99% (data on geographical areas) and 17.48% (data on white wine varieties) of the total variation. With respect to the collection of phytochemical data for wines from certain geographical areas, discrimination of white wine varieties in geographical areas has been observed. Surprisingly, the phytochemical data recorded for wine samples from the Stefanesti geographical area overlapped with those recorded for wine samples from the Dragasani geographical area. This result is surprising, because spectrophotometric analysis allowed the wines from Dragasani and Stefanesti to be discriminated against [38,41]. Taking into account the polyphenolic attributes, the PFT discriminated against the evidence according to the year of production.

Similarities have been observed in wines from the same region, but not in all wine varieties, and this trend has been confirmed. Data obtained from wines from the Silagiu and Blaj wine regions showed similarities, as did Iasi with Cotnari. The clearly distinguished wine-growing regions are Dobrogea (Babadag vineyard), Moldova (the wine-growing center of Iasi and Cotnari vineyards), and the center of Transylvania (the Blaj wine-growing center). The differences between the phytochemical parameters of these wine regions in relation to the wine varieties indicate the extent to which the subgroups have similar characteristics in terms of the catechins and tannins of the wine. The most important factor that presented the main descriptor parameter for the distinction between wine-growing regions was the total polyphenol content.

In red wines (Table 4), in addition to the polyphenolic compounds and anthocyanins, the color intensity and the hue were studied. It is noted that the highest content of polyphenols is found in wines from the Dragasani, Silagiu, and Stefanesti geographical areas, except for some varieties of wine, such as Feteasca Neagra (2908 ± 0.41 mg GAE/L) and Cabernet Sauvignon (2425 ± 0.41 mg GAE/L) from Stefanesti, year of production 2019, which is similar to that in the bibliography [35,36,40]. The content of total anthocyanins for the Merlot 2019 wine from Focsani (179 ± 1.21 mg/L), Feteasca Neagra 2019 from Stefanesti (264 ± 0.71 mg/L), and the Feteasca Neagra 2021 wine (288 ± 0.71 mg/L) are close to those in the bibliography [40,42].

Total polyphenolic compounds, expressed as gallic acid, range from 1194.44 ± 1.01 mg/L to 6482.78 ± 0.03 mg/L. As observed in the bibliography, the highest content of polyphenols is found in wines from the Dragasani, Silagiu, and Stefanesti geographic areas, except for some varieties of wine, such as Feteasca Neagra (2908 ± 0.41 mg GAE/L) and Cabernet Sauvignon (1966 ± 0.41 mg GAE/L) from Stefanesti, year of production 2019, as well as Pinot Verdot 2021 (1778.33 ± 0.26 mg GAE/L) from Dragasani [39,40,43]. In [39,40,42,43], Cabernet Sauvignon has a total polyphenol content of 3377.6 ± 369.6 mg GAE/L, similar to that of Stefanesti in 2021 (3352.77 ± 0.32 mg GAE/L) and Dragasani wine; la Merlot has a total polyphenol content of 3447.5 ± 372.3 mg GAE/L, similar to Dragasani wine (3265.55 ± 0.39 mg GAE/L). The total content of catechins, expressed in catechins, varies between 0.3934 ± 0.01 mg/L and 12.1859 ± 0.01 mg/L (Table 4). The highest value is observed in Burgund Mare 2019 from the Silagiu vineyard and the lowest in the Pinot Noir 2019 variety from the Stefanesti wine center. In [44,45], catechins are in smaller quantities in Cabernet Sauvignon (2.7 × 10^−3^ ± 1.7 × 10^−4^ mol/L) and Si la Merlot (2.2 × 10^−3^ ± 2.0 × 10^−4^ mol/L). The highest total content of tannins can be found in Merlot 2021 (14.473 ± 0.16 mg/L) from the Babadag vineyard and the lowest in Cabernet Sauvignon 2019 from the wine-growing center of Iasi (0.4584 ± 0.04 mg/L). In [43], the content of tannins is much higher in Cabernet Sauvignon, at 79.1 ± 1.7 mg/L, with Merlot containing 79.2 ± 4.2 mg/L and Shiraz containing 75.1 ± 1.7 mg/L; this is also due to the method of determining tannins. Anthocyanins range from 152 ± 0.12 mg/L (Pinot Noir 2019 from the Stefanesti wine center) to 1348 ± 0.12 mg/L (Cabernet Franc from the Dragasani vineyard). In [44], the content of anthocyanins is similar to the studied wine varieties (Table 4), Cabernet Sauvignon (681.8 ± 100.8 mg/L), Merlot (644.1 ± 37.6 mg/L), and Shiraz (301.4 ± 18.9 mg/L). The antioxidant activity is directly proportional to the polyphenolic compounds and is found to be between the values of 52.877 ± 0.02 mg GAE/L (Pinot Noir 2019 from the Stefanesti wine center) and 96.713 ± 0.28 mg GAE/L (Cabernet Franc 2019 from the Silagiu vineyard). In [43], the antioxidant activity of Cabernet Sauvignon is 82.2 mg GAE/L, and the antioxidant activity of Merlot is 68.86 mg GAE/L. The color intensity is a parameter directly proportional to the polyphenolic compounds, and it varies between the values 1.9397 ± 0.00 (Cabernet Sauvignon 2019 from Stefanesti) and 8.4624 ± 0.03 (Merlot 2019 from the Dragasani vineyard). In [44], the intensity of coloring is higher in Cabernet Sauvignon (15.2 ± 0.9) and in Merlot (17.3 ± 0.5). Hue is a parameter directly proportional to the polyphenolic parameters and varies between the values 0.5964 ± 0.04 (Pinot Noir 2019 from the Babadag vineyard) and 1.4003 ± 0.02 (Pinot Noir 2019 from the Stefanesti wine center). In [44], the coloring intensity is higher for Cabernet Sauvignon wine varieties (15.2 ± 0.9), Merlot (17.3 ± 0.5), and Shiraz (5.8 ± 0.1).

Classification and differentiation between samples of red wine based on phenolic compounds was carried out by multivariate data analysis using Principal Component Analysis (PCA). Figure 2 shows a two-dimensional graph of the thirty samples of red wine analyzed, defined by the first two main components, PC1 and PC2. The PC1 main component explains 43.99% of the total variation and opposes the color intensity, tannin hue, and content to the remaining measured parameters.

The second principal component explains PC2 17.48% of the total variation and opposes the color index of anthocyanins with the rest parameters. PCA allows discrimination of wines analyzed in two groups based on the variety (Figure 2a) and the geographical area (Figure 2b). Together, PC1 and PC2 represent 61.48% of the overall variance. Four main groups were identified: the first included the rose wine (Busuioaca de Bohotin from the Tarnave vineyard), the second was from Moldova (the wine-growing center of Iasi and the Cotnari vineyard), and the third included the wines from Transylvania (the Silagiu vineyard). Rose wine was clustered in the negative lower part of PC1 and the positive part of PC2. In the second group on the negative side of PC1 and positive PC2 are the 2019 red wines from Stefanesti and Dragasani, while the wines from Babdag 2021 are in the third group in the upper part of PC2 and in the lower positive part of PC1. Group four can be found in several geographical areas (Silagiu, Tarnave, and Dragasani wines and some from Stefanesti).

**Figure 3:**
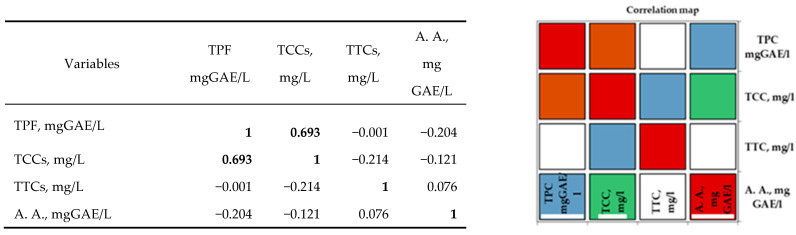
Pearson correlation between the bioactive properties of different white wines. The codes are as follows: (TPC) total phenolic content, (TCCs) total catechin compounds, (TTCs) total tannic compounds, and (A.A.) antioxidant activity.

In the case of white varieties, Pearson’s correlation analysis (Figure 3) shows positive correlations between the bioactive properties of the investigated grape musts and their corresponding wines. All the correlation coefficients were higher than 0.5. A strong positive correlation was observed for antioxidant activity, with the total tannin content and total catechin content demonstrating the importance of these two categories of parameters in perfecting the taste and flavor of white wines. Additionally, a strong correlation was observed between the TCCs and TTCs of the white musts and wines.

Analysis of the Pearson correlation (Figure 3) shows a strong positive correlation between total polyphenolic compounds and white wine catechin content in the studied geographical areas. Between the antioxidant activity and the tannin content, there is a moderate positive correlation. Correlations between the other compounds are moderate negative correlations. The interpretation of the correlation analysis was performed using correlation coefficients with values higher than 0.5.

**Figure 4:**
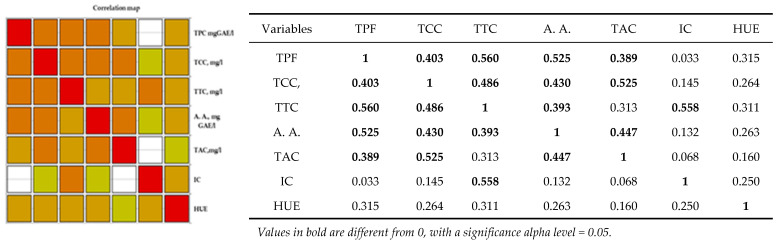
Pearson correlation maps of coefficients of determination (Pearson) on red wine: The codes are as follows: (CI) color intensity, (HUE), (TPC, mg GAE/L) total phenolic content, (TCCs, mg/L) total catechin compounds, (TTCs, mg/L) total tannic compounds, (TACs, mg/L) total anthocyanin compounds, and (A.A., mgGAE/L) antioxidant activity.

Correlation maps of coefficients of determination (Pearson) on white wine are shown below:

Analysis of the Pearson correlation (Figure 4) shows a positive correlation between all polyphenolic compounds and the color of wine, including the antioxidant activity of red wine. There is a strong positive correlation between total polyphenolic compounds and the content of catechins, tannins, anthocyanins, and antioxidant activity in red wines from the studied geographical areas. Between antioxidant activity and the content of tannins, catechins, and anthocyanins, there is a strong positive correlation between the content of anthocyanins, catechins, and antioxidant activity. There is a strong positive correlation between the intensity of the color of red wine and the total tannin content. Correlations between wine and hue are moderately positive correlations.

Correlation maps of coefficients of determination (Pearson) on red wine are shown below:

Statistical tests confirmed significant differences in the polyphenolic parameters of the selected red wines in relation to the wine regions. The areas drawn with white in Figure 4 are significant differences between the parameters mentioned in the table. As shown in Figure 4, the orange colors are the strong correlations mentioned in the table.

### 3.2. Individual Phenolic Compounds in White and Red Wine by UHPLC–ESI/HRMS

**Figure 5:**
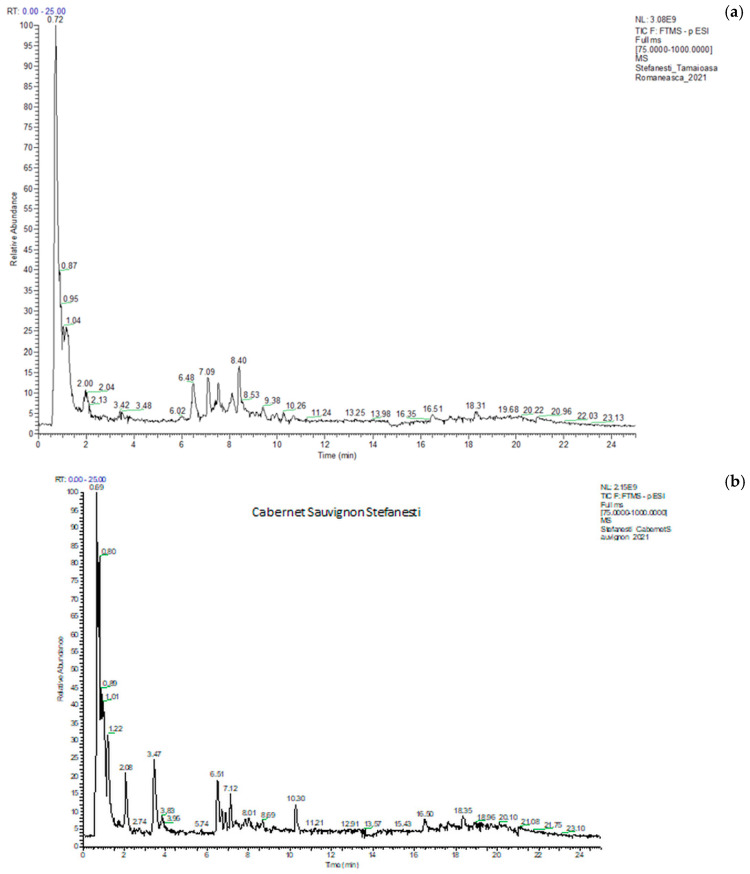
Chromatograms of Tamaioasa Romaneasca (**a**) wine and Cabernet Sauvignon (**b**) from Stefanesti region in 2021.

The identification and quantification of polyphenolic compounds were carried out by UHPLC–ESI/HRMS analysis using an external calibration method [31]. A typical chromatogram of the total ion current (TIC) is shown in Figure 5 (a) Tamaioasa Romaneasca 2021 and (b) Cabernet Sauvignon 2021, both from Stefanesti. The retention time, compound name, formula, m/z values of adduct ions and MS/MS fragment ions in negative ESI mode, mass error, and the exact molecular mass are given in S1. The analysis was conducted in duplicate, and the results were expressed as mean values and standard deviations.

The HPLC analysis obtained a total of 13 phenolic compounds (Table 5) from 25 white wines and 23 polyphenolic compounds (Table 6) from 24 red wines, and they have been simultaneously identified and quantified by comparison with reference standards, including phenolic acids, flavonoids, stilbens (t-resveratrol), plant hormone (abscisic acid), ellagic acid, and, a dimeric derivative of gallic acid, esters of caffeic acid (chlorogenic acid and CAFE—a powerful bioactive compound).

The results obtained on the polyphenolic profile of white wines, as shown in Table 5, are that wine of the Aligote variety has a gallic acid content similar to that found in the bibliography, 10.93 ± 0.02 mg/L [38,41,46], which is similar to that of Riesling Italian, which is a little smaller than found in the bibliography [38,41,46]. As for 3,4-dihi-droxybenzoic acid in the Chardonnay from 2021 (0.71 ± 0.03 mg/L), it is similar to the content expressed in the bibliography [38,41,46] and a little lower than Chardonnay 2019, 0.20 ± 0.00 mg/L. In the case of catechin, the white varieties visualized in Table 6 are lower than the values expressed in the bibliography [38,41,46]. The catechin values are between 0.61 ± 0.03 mg/L in Aligote 2021 from Babadag and 2.31 ± 0.29 mg/L in Chardonnay 2021 wine from Dragasani; exceptions are Feteasca Regala 2021 from Stefanesti and Caramposie Selected 2021 from Dragasani (0.04 ± 0.00 mg/L). In the same situation, there is the epi-catechin content, which is very small compared to the bibliography [38,41,46]. The syringic acid is similar to levels found in the bibliography [38,41,46]; the lowest value is noted in Pinot Gris 2019 from Babadag (3.45 ± 0.08 mg/L), followed by Cramposie Selectionata 2021 from Dragasani (4.88 ± 0.07 mg/L); average values of this acid are found in Muscat Ottonel 2021 from Stefanesti (42.51 ± 1.25 mg/L), and superior values of syringic acid are found in Aligote 2021 from Babadag (103.71 ± 2.12 mg/L) and Feteasca Regala 2019 from Tarnave (96.13 ± 2.54 mg/L). Chardonnay 2019 (0.70 ± 0.21 mg/L) and 2021 (0.62 ± 0.03 mg/L) from Babadag have the same ferulic acid content as in the bibliography [38,41,46].

The content of resveratrol in white wines (Table 5) is noted to be included in the values expressed in the bibliography [41,46]. The lowest resveratrol content in Table 5 is found in the Chardonnay 2021 wine variety (0.07 ± 0.03 mg/L) from Babadag, Feteasca Regala 2021 (0.02 ± 0.00 mg/L) from Stefanesti, and Sauvignon Blanc 2021 (0.01 ± 0.00 mg/L) and Muscat Ottonel 2021 (0.03 ± 0.00 mg/L) from Stefanesti. It is noted that most of the white wines from Stefanesti have a very low resveratrol content compared to the other wines studied and highlighted in Table 5. The highest content of white wine is found in Feteasca Regala 2021 (0.36 ± 0.02 mg/L) from Dragasani and Riesling Italian (0.55 ± 0.07 mg/L) 2019 from Stefanesti, which shows that both the geographical area and the year of production, as well as the climate of that year, are important for the resveratrol content of the wine.

Another polyphenolic biocompound with antioxidant activity is quercetin, which is a flavonol; the levels of this compound in white wine varieties fall within the values described in the bibliography [37,38,45]. Lower values for quercetin can be found in Chardonnay 2021 (0.0013 ± 0.11 mg/L) from Babadag and in Sauvignon Blanc 2019 (0.0011 ± 0.77 mg/L) from Stefanesti.

In the case of red wines, in Table 6, they have a gallic acid content similar to that in the bibliography [37]. The gallic acid content of red wines varies between 3.86 ± 0.25 mg/L (Cabernet Sauvignon 2019 from Dragasani) and 155.4 ± 1.74 mg/L (Merlot 2019 from Babadag). In red wines, high amounts of catechins and epi-catechins are recorded, which fall within the values expressed in the bibliography [45,46]. The catechin content with the highest value is noted in Cabernet Sauvignon 2021 from Dragasani (130.99 ± 0.7 mg/L), and this wine is noted as having the highest epi-catechin content (87.661 ± 1.42 mg/L). The content of syringic acid is much higher in red wines, and the values are high compared to the bibliography [37,44,46]. The values of syringic acid, noted in Table 6, range from 57.34 ± 1.41 mg/L (Cabernet Sauvignon 2019 from Stefanesti) to 3129.48 ± 70.7 mg/L (Burgund 2019 from Silagiu). One of the important compounds in red wine is resveratrol, which can reach values of 7.409 ± 0.05 mg/L in Negru de Dragasani 2021 and Feteasca Neagra 2021 from Stefanesti (7.245 ± 0.52 mg/L), much higher than found in the bibliography [37,44,46].

**Figure 6:**
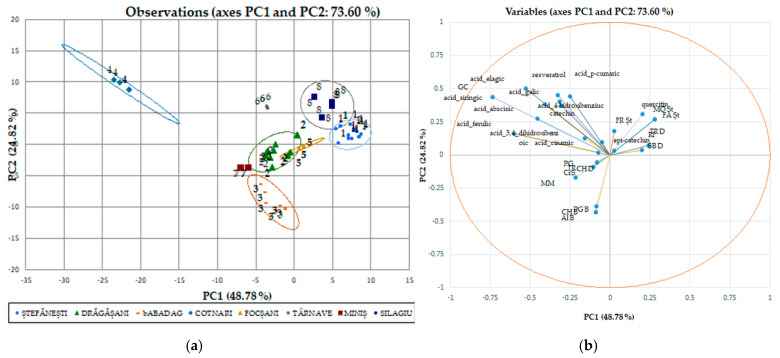
White wine statistical models using discriminatory analysis (DA), a supervised method for individual polyphenol score plots discriminated by geographical areas (**a**) and varieties (**b**).

We used discriminant analysis (DA), which involves the derivation of some canonical variables that can explain interclass variation in a similar manner to that of PCA, for individual polyphenols performed separately from the red wines (Figure 6a,b) due to the major difference between their phytocompounds. Although PCA allows the classification of red wines by variety, it cannot identify wines by geographical origin. The PCA method does not take into account the relationship between certain groups, but it selects a retaining direction, i.e., the maximum structure in a smaller size. Unlike PCA, DA is a method of recognizing the supervised model in wine analysis, i.e., pattern recognition, that is, data grouping [47]. The discriminant analysis of polyphenolic composition of Romanian white wines has been applied separately to the two collections of phytochemicals. The first two axes (shown in the diagram) explain 73.60% of the total variation in the phytochemical composition. The maps defined by PC1 and PC2 for the DA of the polyphenolic data of the white wine varieties analyzed are shown in Figure 6a and Figure 6b, respectively. The two main components took into account 48.78% (data on geographical areas) and 24.82% (data on white wine varieties) of the total variation. The collection of phytochemical data for Romanian wines was in favor of the discrimination of white wine varieties by geo-graphic areas. Surprisingly, the phytochemical data recorded for wine samples from the Stefanesti geographical area overlap with those recorded for wine samples from the Dragasani geographical area.

Taking into account the polyphenolic attributes, the individual polyphenols were discriminated against the evidence according to geographical origin and year of production. As seen from the Pearson matrix (Table S3), the gallic acid in white wines is strongly correlated with 3,4,DHBA, 4-HBA, catechin, siringic acid, p-coumaric acid, ferulic acid, resveratrol, ellagic acid, abscisic acid, and quercetin. Similarly with red wines (Table S4), gallic acid strongly correlates with the same compounds, except for the ellagic acid with which it is weakly negatively correlated (−0.165). The same polyphenolic acid is also strongly correlated with naringin (0.427) and CAPE (0.823).

**Figure 7:**
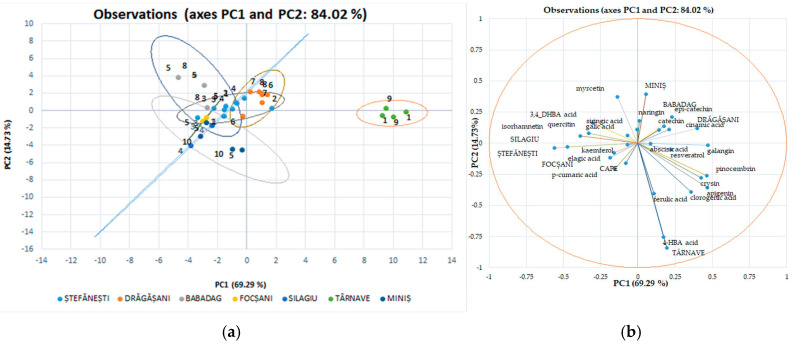
Red wine statistical models using discriminatory analysis (DA), a supervised method for individual polyphenol score plots discriminated by geographical areas (**a**) and varieties (**b**).

In Figure 7, the associated individual polyphenolic compounds indicate the variables that significantly influenced the rearrangement of data in the new axis system. Among the analyzed polyphenolic compounds, chlorogenic acid, gallic acid, resveratrol, epi-catechin, ferulic acid, and 4, HBA had a major impact on the first main component of PC1; while catechin, epi-catechin, and cinnamic acid strongly influenced the second main component of PC2. These observations show that the ability to discriminate against polyphenols to classify the studied evidence has certain limitations. Using this method of discrimination on individual polyphenolic compounds in red wines, the variation for separation resulted in 84.02%; for main component 1, this value is 69.29%, and for main component 2, it is 14.73%, with significant differences between wine varieties and geographical areas.

## 4. Conclusions

Our results provide the largest database available to date on polyphenol, anthocyanin, and antioxidant activity and the individual profile content of Romanian white and red wines. Polyphenolic compounds are important for classifying wines. Cinnamic acids, catechin and epi-catechin, resveratrol, gallic acid, as well as procyanidins, are compounds that show considerable differences between wine varieties. Differences in the quality and quantity of polyphenols could be caused by varietal differences in wines and could be used to interpret the genetic differences in grapes used in winemaking. Polyphenolic compounds can differ significantly from year to year, especially as a result of climatic and environmental factors.

The statistical analysis of wines showed strong coordination between polyphenolic compounds, antioxidant activity, and the individual profiles of polyphenols. This can potentially be used as a wine authentication system. The statistical evaluation and the discrimination of wine samples by DA confirmed the strong link between individual polyphenolic compounds and wine varieties. Thus, the differentiation of wines with the help of polyphenolic compounds can be completed on the variety of wines and terroir.

## Figures and Tables

**Table 1:** Description of white wines (variety, geographical region and producer, year of production, and code).

The Wine White Varieties	Symbol	Geographic Region	Vineyard/Wine Center	Year of Production
Riesling Italian	RI19/21_St	Muntenia	Stefanesti	2019, 2021
	RI19_Bj	Transilvania	Blaj	2019
Feteasca Regala	FR21_St	Muntenia	Stefanesti	2021
	FR21_D	Oltenia	Dragasani	2021
	FR21_T	Transilvania	Tarnave	2019
Sauvignon Blanc	SB19/21_St	Muntenia	Stefanesti	2019, 2021
	SB19_D	Oltenia	Dragasani	2019
	SB19_IS	Northern Moldova	Iasi	2019
	SB19_F	Southern Moldova	Focsani	2019
Pinot Gris	PG19/21_B	Dobrogea	Babadag	2019, 2021
Muscat Ottonel	MO19/21_St	Muntenia	Stefanesti	2019, 2021
	MO19_Bj	Transilvania	Blaj	2019
	MO19_S	Banat	Silagiu	2019
Riesling de Rihn	RR19_D	Oltenia	Dragasani	2019
Cramposie Selecționata	CrS19/21_D	Oltenia	Dragasani	2019, 2021
Feteasca Alba	FA19/21_St	Muntenia	Stefanesti	2019, 2021
	FA19_IS	Northern Moldova	Iasi	2019
	FA19_S	Banat	Silagiu	2019
Tamaioasa Romaneasca	TR21_St	Muntenia	Stefanesti	2021
	TR19/21_D	Oltenia	Dragasani	2019, 2021
Aligote	Al19/21_B	Dobrogea	Babadag	2019, 2021
Chardonnay	CH19/21_B	Dobrogea	Babadag	2019, 2021
	CH21_D	Muntenia	Dragasani	2021
Traminer Roz	TR R19_Bj	Transilvania	Blaj	2019
Mustoasa de Moderat	MM19_M	Banat	Minis	2019
Grasa de Cotnari	GC19_C	Northern Moldova	Cotnari	2019

**Table 2:** Description of red wines (variety, geographical region and producer, year of production, and code).

The Wine Red Varieties	Symbol	Geographic Region	Vineyard/Wine Center	Year of Production
Burgund Mare	BM19/21_St	Muntenia	Stefanesti	2019, 2021
	BM19_Bj	Transilvania	Blaj	2019
	BM19_S	Banat	Silagiu	2019
Cabernet Sauvignon	CS19/21_St	Muntenia	Stefanesti	2019, 2021
	CS19_D	Oltenia	Dragasani	2019, 2021
	CS19_IS	Northern Moldova	Iasi	2019
Feteasca Neagra	FN19/21_St	Muntenia	Stefanesti	2019, 2021
	FN19_C	Northern Moldova	Cotnari	2019
	FN19_D	Oltenia	Dragasani	2019
Merlot	M21_St	Muntenia	Stefanesti	2019, 2021
	M19_IS	Northern Moldova	Iasi	2019
	M19_Foc	Southern Moldova	Focsani	2019
	M19/21_Bab	Dobrogea	Babadag	2019, 2021
Pinot Noir	PN19/21_St	Muntenia	Stefanesti	2019, 2021
	PN19/21_B	Dobrogea	Babadag	2019, 2021
Negru de Dragasani	ND19/21_D	Oltenia	Dragasani	2019, 2021
Cabernet Franc	CF19/21_D	Oltenia	Dragasani	2019, 2021
	CF19_Bj	Transilvania	Blaj	2019
	CF19_S	Banat	Silagiu	2019
Cadarca	CD19_M	Banat	Minis	2019
Pinot Verdot	PV19/21_D	Oltenia	Dragasani	2019, 2021

**Table 3:** Polyphenolic compounds in white wines.

Varieties White Wine	TPF mgGAE/L	TCCs, mg/L	TTCs, mg/L	A. A., mgGAE/L
A21_B	330.22 ± 1.414	0.0552 ± 0.0014	7.324 ± 0.063	54.837 ± 0.283
A19_B	240.22 ± 0.364	0.2153 ± 0.004	6.4365 ± 0.0064	65.705 ± 0.877
CH19_B	334.29 ± 0.827	0.2882 ± 0.002	8.6065 ± 0.003	71.986 ± 0.162
CH21_B	397.89 ± 1.351	0.1696 ± 0.0011	15.1819 ± 0.349	70.47 ± 0.389
PG19_B	734.07 ± 0.735	0.3524 ± 0.0055	8.8862 ± 0.066	53.93 ± 0.354
PG21_B	1349.44 ± 1.17	0.8937 ± 0.024	9.0982 ± 0.081	56.34 ± 0.827
FA19_S	871 ± 0.707	1.2541 ± 0.003	0.5271 ± 0.001	27.341 ± 0.158
MO19_S	1058 ± 1.192	1.5728 ± 0.009	0.4798 ± 0.004	38.74 ± 0.361
RR19_D	644.4 ± 1.534	0.1061 ± 0.005	n.d.	73.073 ± 0.123
SB19_D	293.07 ± 0.127	0.4787 ± 0.001	n.d.	50.645 ± 0.275
CH21_D	542 ± 1.414	0.3897 ± 0.001	0.0051 ± 0.000	58.27 ± 0.318
CrS19_D	703.05 ± 0.120	0.4957 ± 0.001	n.d.	58.35 ± 0.375
CrS21_D	512 ± 1.778	0.4107 ± 0.023	0.0045 ± 0.001	51.56 ± 1.103
FR21_D	328 ± 1.577	0.6262 ± 0.009	0.0023 ± 0.000	50.99 ± 0.190
TR19_D	730.85 ± 1.697	0.5534 ± 0.002	n.d.	52.89 ± 0.163
TR21_D	560.33 ± 0.636	0.1248 ± 0.003	1.76421 ± 0.008	50.16 ± 0.608
FA19_IS	528 ± 0.707	1.3214 ± 0.006	n.d.	34.562 ± 0.146
SB19_IS	693 ± 0.707	1.4102 ± 0.014	n.d.	38.12 ± 0.226
SB19_F	314 ± 0.708	0.7152 ± 0.000	0.2741 ± 0.000	31.135 ± 0.071
FA19_St	245.848 ± 0.75	0.1705 ± 0.001	n.d.	20.183 ± 0.633
FA21_St	730.17 ± 1.52	0.1328 ± 0.014	n.d.	49.54 ± 1.089
FR21_St	402.56 ± 1.208	0.1534 ± 0.004	1.6979 ± 0.019	53.238 ± 0.177
MO19_St	455.96 ± 0.565	0.1317 ± 0.015	n.d.	28.086 ± 0.169
MO21_St	429 ± 0.313	0.163 ± 0.003	1.4028 ± 0.018	73.88 ± 0.778
RI19_St	394 ± 1.414	0.501 ± 0.001	n.d.	54.32 ± 0.283
RI21_St	392 ± 0.364	0.0863 ± 0.004	0.91908 ± 0.006	73.83 ± 0.878
SB19_St	273.292 ± 1.199	0.6379 ± 0.004	n.d.	24.678 ± 0.235
SB21_St	983 ± 0.536	0.1248 ± 0.011	1.76421 ± 0.010	78.97 ± 0.693
TR21_St	560.33 ± 0.325	0.1248 ± 0.007	1.7621 ± 0.006	59.822 ± 0.123
RI19_Bj	1156 ± 0.949	1.5077 ± 0.008	n.d.	34.78 ± 0.481
MO19_Bj	1183 ± 0.536	2.1548 ± 0.002	n.d.	38.14 ± 0.064
Trm19_Bj	1086 ± 0.657	2.0119 ± 0.007	n.d.	37.59 ± 0.198
FR19_T	586 ± 0.535	0.2654 ± 0.003	n.d.	51.745 ± 0.008
GC19_C	682 ± 0.707	0.5247 ± 0.007	n.d.	36.63 ± 0.247
MM21_M	364 ± 0.414	0.1031 ± 0.005	0.062 ± 0.006	50.2 ± 0.862

n.d.—not determined; TPFs—total polyphenol compounds, TCCs—total catechin compounds, TTCs—total tannic compounds and A.A.—antioxidant activity; each value represents the mean standard deviation.

**Table 4:** Polyphenolic compounds in red wines.

Varieties Red Wine	TPF mgGAE/L	TCC, mg/L	TTC, mg/L	A. A., mg GAE/L	TAC, mg/L	CI DO (420 + 520 + 620)	H, DO (420/520)
M19_B	3432.017 ± 0.01	2.0602 ± 0.04	3.437 ± 0.00	95.24 ± 0.49	502 ± 1.41	8.2474 ± 0.35	0.7225 ± 0.02
M21_B	3754.44 ± 1.10	3.951 ± 0.03	14.473 ± 0.16	77.47 ± 0.29	670 ± 1.12	4.484 ± 0.03	0.8721 ± 0.01
PN19_B	2372.57 ± 0.40	1.0183 ± 0.00	6.578 ± 0.01	88.63 ± 0.18	247 ± 1.80	4.6434 ± 0.00	0.5964 ± 0.04
PN21_B	1194.44 ± 1.01	4.2415 ± 0.00	6.5108 ± 0.00	78.64 ± 0.29	329 ± 1.83	6.0033 ± 0.23	0.9853 ± 0.00
BM19_S	4166 ± 1.41	12.1859 ± 0.01	2.2969 ± 0.00	94.4753 ± 0.03	488 ± 1.82	8.3093 ± 0.06	1.1139 ± 0.01
CF19_S	4353 ± 0.12	9.3643 ± 0.02	4.8874 ± 0.04	96.713 ± 0.28	615 ± 1.12	7.9329 ± 0.04	1.0244 ± 0.01
CS19_D	4234.79 ± 0.12	1.5161 ± 0.06	1.4553 ± 0.00	89.78 ± 0.64	299 ± 0.71	5.9414 ± 0.03	1.0504 ± 0.00
CS21_D	3320 ± 1.41	2.973 ± 0.01	2.7842 ± 0.02	88.32 ± 0.4	312 ± 0.82	7.2103 ± 0.02	1.2622 ± 0.02
M19_D	3265.55 ± 0.39	5.7645 ± 0.02	2.9246 ± 0.01	89.02 ± 0.07	405 ± 1.41	8.4624 ± 0.03	1.0226 ± 0.01
ND21_D	4043.33 ± 0.14	9.4319 ± 0.00	9.4319 ± 0.02	94.23 ± 2.16	1023 ± 1.82	3.6919 ± 0.16	0.9602 ± 0.17
ND19_D	3638.13 ± 0.18	1.615 ± 0.01	4.6127 ± 0.4	96.67 ± 0.06	605 ± 0.80	7.7018 ± 0.01	1.0385 ± 0.03
CS19_IS	1966 ± 0.41	0.5454 ± 0.02	0.4584 ± 0.04	65.76 ± 0.39	293 ± 0.13	1.143 ± 0.01	0.835 ± 0.02
M19_IS	2198 ± 0.54	0.7129 ± 0.00	0.5463 ± 0.00	72.54 ± 0.3	288 ± 0.25	0.8474 ± 0.00	0.7982 ± 0.01
M19_F	2707 ± 0.12	5.0957 ± 0.00	2.0707 ± 0.00	54.32 ± 0.01	179 ± 1.21	6.565 ± 0.01	1.0978 ± 0.00
BM19_St	3401 ± 0.71	0.8954 ± 0.00	1.944 ± 0.00	67.335 ± 0.01	279 ± 1.41	3.6855 ± 0.00	0.8581 ± 0.00
BM21_St	4216.67 ± 0.37	3.675 ± 0.01	5.4127 ± 0.01	85.676 ± 0.16	291.9 ± 0.72	6.3092 ± 0.00	0.9794 ± 0.00
CS19_St	2425 ± 0.41	0.4012 ± 0.00	2.1215 ± 0.00	56.33 ± 0.1	280 ± 1.82	1.9397 ± 0.00	1.1005 ± 0.01
CS21_St	3352.77 ± 0.32	5.5494 ± 0.00	9.8742 ± 0.00	85.21 ± 0.08	608.0 ± 40.05	6.5856 ± 0.01	1.3081 ± 0.00
FN19_St	2908 ± 0.41	0.656 ± 0.00	1.369 ± 0.00	59.669 ± 0.01	264 ± 0.71	4.1793 ± 0.00	0.8402 ± 0.00
FN21_St	3027 ± 0.12	5.1286 ± 0.00	4.4721 ± 0.00	62.46 ± 0.01	288 ± 0.71	4.8963 ± 0.00	1.2375 ± 0.01
M21_St	5212.78 ± 0.03	4.4725 ± 0.00	4.2327 ± 0.00	90.78 ± 0.33	670 ± 0.83	4.6839 ± 0.00	0.8586 ± 0.01
PN19_St	2217.02 ± 0.53	0.3934 ± 0.01	0.8727 ± 0.00	52.877 ± 0.02	152 ± 0.12	2.3136 ± 0.01	1.4003 ± 0.02
PN21_St	6482.78 ± 0.03	7.8342 ± 0.01	4.3348 ± 0.00	88.07 ± 0.57	1391 ± 0.82	5.852 ± 0.02	1.1846 ± 0.01
CD19_M	2786 ± 0.41	9.4751 ± 0.02	4.5865 ± 0.02	70.236 ± 0.09	936 ± 2.81	6.4177 ± 0.05	0.9802 ± 0.04
FN19_C	3339 ± 0.12	4.2813 ± 0.01	1.9515 ± 0.00	87.945 ± 0.03	240 ± 0.82	5.9924 ± 0.01	0.8365 ± 0.02
PN19_T	2132.22 ± 0.55	5.012 ± 0.56	1.898 ± 0.01	85.44 ± 0.32	453 ± 0.53	3.935 ± 0.13	1.1846 ± 0.04
PV19_D	2397.78 ± 0.65	4.0028 ± 0.01	1.6648 ± 0.00	72.87 ± 0.016	1032 ± 1.12	7.3098 ± 0.04	0.9545 ± 0.02
PV21_D	1778.33 ± 0.26	2.249 ± 0.14	1.1614 ± 0.01	86.32 ± 0.54	871 ± 0.41	4.5309 ± 0.01	0.9492 ± 0.01
CF19_D	3271.461 ± 0.5	1.1571 ± 0.00	2.2278 ± 0.01	82.28 ± 0.31	294 ± 0.83	7.2798 ± 0.01	1.3173 ± 0.01
CF21_D	2103.89 ± 0.12	3.2554 ± 0.01	0.731 ± 0.00	85.77 ± 0.18	1348 ± 0.12	7.4103 ± 0.19	0.7617 ± 0.01

TPF—total polyphenol compounds, TCCs—total catechin compounds, TTCs—total tannic compounds, TACs—total anthocyanin compounds, A.A.—antioxidant activity, CI—color intensity, and H—color hue; each value represents the mean standard deviation.

**Table 5:** Concentrations of each phenolic compound found in the examined samples. Results are given in mg/L ± standard deviation. Mean value of duplicate extraction assays.

Varieties White Wine	Gallic Acid	3,4,DHBA	4,HBA	Catechin	Epi-Catechin	Siringic Acid	p-Coumaric Acid	Ferulic Acid	Resveratrol	Ellagic Acid	Abscisic Acid	Cinnamic Acid	Quercetin
Al_B_21	10.93 ± 0.02	1.73 ± 0.01	n.d.	0.61 ± 0.03	0.06 ± 0.015	103.71 ± 2.12	0.32 ± 0.01	0.74 ± 0.43	0.19 ± 0.04	0.12 ± 0.02	0.29 ± 0.02	0.21 ± 0.04	0.0807 ± 1.88
CH_B_19	1.81 ± 0.06	0.20 ± 0.00	n.d.	1.67 ± 0.02	0.34 ± 0.01	15.06 ± 1.41	0.56 ± 0.23	0.70 ± 0.21	0.07 ± 0.03	0.12 ± 0.03	0.12 ± 0.01	0.02 ± 0.03	0.0129 ± 0.23
CH_B_21	1.18 ± 0.03	0.71 ± 0.03	n.d.	1.41 ± 0.05	0.63 ± 0.01	7.80 ± 0.31	0.31 ± 0.04	0.62 ± 0.03	0.41 ± 0.00	0.06 ± 0.00	0.10 ± 0.01	1.15 ± 0.00	0.0005 ± 0.04
PG_B_19	2.10 ± 0.01	0.75 ± 0.00	n.d.	1.86 ± 0.02	0.36 ± 0.00	12.83 ± 0.01	0.17 ± 0.00	0.69 ± 0.01	0.38 ± 0.00	0.10 ± 0.00	0.12 ± 0.00	0.47 ± 0.01	0.0013 ± 0.11
PG_B_21	0.58 ± 0.01	0.20 ± 0.01	n.d.	0.91 ± 0.39	0.70 ± 0.14	3.45 ± 0.08	0.61 ± 0.18	0.10 ± 0.03	0.11 ± 0.001	0.01 ± 0.00	0.09 ± 0.05	0.05 ± 0.00	0.0013 ± 0.11
FA_S_19	3.59 ± 0.51	n.d.	0.08 ± 0.005	0.45 ± 0.23	0.11 ± 0.01	28.34 ± 0.31	0.30 ± 0.03	0.03 ± 0.01	0.25 ± 0.16	0.06 ± 0.01	0.04 ± 0.01	0.03 ± 0.00	0.2467 ± 3.02
MO_S_19	6.70 ± 0.19	0.89 ± 0.07	0.12 ± 0.04	0.70 ± 0.07	0.16 ± 0.01	64.27 ± 1.71	0.16 ± 0.02	0.07 ± 0.01	0.27 ± 0.01	0.17 ± 0.02	0.02 ± 0.00	0.01 ± 0.00	0.2657 ± 3.65
CH_D_21	2.05 ± 0.16	0.20 ± 0.01	0.05 ± 0.00	4.45 ± 0.05	2.31 ± 0.29	18.75 ± 1.03	0.06 ± 0.01	0.05 ± 0.00	0.14 ± 0.03	0.02 ± 0.00	0.02 ± 0.00	0.07 ± 0.00	0.0011 ± 0.10
CrS_D_21	0.47 ± 0.08	0.06 ± 0.14	0.04 ± 0.00	0.04 ± 0.00	n.d.	4.88 ± 0.07	0.10 ± 0.00	0.03 ± 0.00	0.12 ± 0.01	0.02 ± 0.00	0.01 ± 0.00	0.01 ± 0.00	0.0012 ± 0.13
FR_D_21	3.53 ± 0.22	0.33 ± 0.04	0.04 ± 0.01	3.16 ± 0.49	2.27 ± 0.44	36.08 ± 0.91	0.09 ± 0.01	0.04 ± 0.00	0.36 ± 0.02	0.05 ± 0.00	0.02 ± 0.00	0.05 ± 0.01	0.0419 ± 0.99
TR_D_21	3.21 ± 0.19	0.25 ± 0.02	0.06 ± 0.00	0.47 ± 0.02	0.30 ± 0.02	31.03 ± 1.31	0.11 ± 0.00	0.04 ± 0.00	0.06 ± 0.00	0.03 ± 0.00	0.02 ± 0.00	n.d.	0.0085 ± 0.19
S_F_19	3.33 ± 0.34	0.73 ± 0.04	0.05 ± 0.00	2.17 ± 0.36	1.53 ± 0.12	31.68 ± 1.31	0.18 ± 0.02	0.02 ± 0.00	0.25 ± 0.01	0.06 ± 0.01	0.02 ± 0.00	0.03 ± 0.00	0.0995 ± 2.08
FA_SF_19	1.59 ± 0.13	0.04 ± 0.00	0.07 ± 0.01	1.29 ± 0.05	1.27 ± 0.11	11.32 ± 0.46	1.34 ± 0.02	0.40 ± 0.03	0.22 ± 0.02	0.05 ± 0.00	0.10 ± 0.05	1.89 ± 0.04	0.0013 ± 0.17
FA_SF_21	1.44 ± 0.38	0.41 ± 0.05	0.11 ± 0.06	0.08 ± 0.01	0.02 ± 0.00	9.69 ± 0.18	2.13 ± 0.06	0.19 ± 0.02	0.06 ± 0.01	0.16 ± 0.02	0.15 ± 0.02	0.09 ± 0.01	0.0032 ± 0.88
FR_SF_21	2.24 ± 0.08	0.93 ± 0.02	0.07 ± 0.01	0.04 ± 0.00	0.01 ± 0.00	15.63 ± 0.99	1.12 ± 0.03	0.35 ± 0.02	0.02 ± 0.00	0.12 ± 0.01	0.14 ± 0.01	0.06 ± 0.00	0.0022 ± 0.30
MO_SF_19	2.22 ± 0.46	0.67 ± 0.22	0.03 ± 0.00	0.79 ± 0.03	0.26 ± 0.03	18.48 ± 0.68	0.10 ± 0.01	0.02 ± 0.00	0.03 ± 0.00	0.12 ± 0.05	0.05 ± 0.00	n.d.	0.2564 ± 3.44
MO_SF_21	4.24 ± 0.12	0.59 ± 0.02	0.11 ± 0.01	3.01 ± 0.58	1.23 ± 0.15	42.51 ± 1.25	0.13 ± 0.02	0.02 ± 0.00	0.35 ± 0.01	0.09 ± 0.00	0.02 ± 0.00	0.01 ± 0.00	0.5124 ± 4.05
R_SF_19	3.22 ± 0.22	0.42 ± 0.02	0.04 ± 0.00	0.85 ± 0.12	0.45 ± 0.02	38.82 ± 1.48	0.11 ± 0.00	0.02 ± 0.00	0.55 ± 0.07	0.03 ± 0.00	0.03 ± 0.00	0.01 ± 0.00	0.2492 ± 2.85
R_SF_21	1.66 ± 0.21	0.05 ± 0.00	0.07 ± 0.02	3.43 ± 0.23	2.98 ± 0.76	17.66 ± 0.65	1.33 ± 0.41	0.22 ± 0.02	0.06 ± 0.01	0.01 ± 0.00	0.13 ± 0.05	0.24 ± 0.04	0.0024 ± 1.22
S_SF_19	2.05 ± 0.18	0.29 ± 0.06	0.04 ± 0.00	0.47 ± 0.05	0.22 ± 0.00	20.56 ± 0.87	0.10 ± 0.00	0.03 ± 0.00	0.33 ± 0.03	0.05 ± 0.00	0.02 ± 0.00	0.01 ± 0.00	0.0352 ± 0.75
S_SF_21	0.82 ± 0.08	0.19 ± 0.01	0.06 ± 0.02	1.75 ± 0.13	0.89 ± 0.21	7.03 ± 0.23	0.53 ± 0.20	0.26 ± 0.08	0.01 ± 0.00	0.01 ± 0.00	0.14 ± 0.03	0.30 ± 0.08	0.0011 ± 0.77
TR_SF_21	3.07 ± 0.44	0.12 ± 0.06	0.06 ± 0.03	1.73 ± 0.27	0.54 ± 0.08	19.76 ± 1.06	0.18 ± 0.09	0.03 ± 0.00	0.28 ± 0.05	0.22 ± 0.06	0.03 ± 0.00	0.03 ± 0.00	0.5307 ± 3.88
FR_T_19	1.09 ± 0.07	0.33 ± 0.08	0.08 ± 0.03	0.37 ± 0.04	0.10 ± 0.00	13.03 ± 0.88	0.52 ± 0.19	0.34 ± 0.10	0.08 ± 0.03	0.49 ± 0.12	0.08 ± 0.01	0.64 ± 0.10	0.0014 ± 0.14
GC_CT_19	6.78 ± 0.72	0.17 ± 0.01	0.03 ± 0.00	0.46 ± 0.05	0.14 ± 0.00	96.13 ± 2.54	0.55 ± 0.22	0.16 ± 0.04	0.15 ± 0.12	0.13 ± 0.05	0.10 ± 0.04	0.04 ± 0.00	0.0006 ± 0.04
MM_T_19	0.99 ± 0.04	0.21 ± 0.02	0.05 ± 0.00	0.64 ± 0.06	0.31 ± 0.10	8.90 ± 0.56	0.22 ± 0.11	0.29 ± 0.09	0.05 ± 0.02	0.10 ± 0.01	0.03 ± 0.00	0.12 ± 0.04	0.0453 ± 0.95

n.d.—not determined; 3,4,DHBA—3,4 dihydroxybenzoic acid; 4,HBA—4,hydroyibenzoic acid.

**Table 6:** Concentrations of each phenolic compound found in the examined samples. Results are given in mg/L ± standard deviation. Mean value of duplicate extraction assays.

Varieties Red Wine	Gallic Acid	3,4,DHBA	4,HBA	Catechin	Chlorogenic Acid	Epi-catechin	Siringic Acid	p-Coumaric Acid	Ferulic Acid	Naringin	Resveratrol	Ellagic Acid	Myricetin	Abscisic Acid	Cinnamic Acid	Quercetin	Pinocembrin	Kaempferol	Iso-Rhamnetin	Apigenin	CAPE	Crysin	Galangin
	mg/L	mg/L	mg/L	mg/L	mg/L	mg/L	mg/L	mg/L	mg/L	mg/L	mg/L	mg/L	mg/L	mg/L	mg/L	mg/L	mg·10^−3^/L	mg·10^−3^/L	mg·10^−3^/L	mg·10^−3^/L	mg·10^−3^/L	mg·10^−3^/L	mg·10^−3^/L
M_B_19	155.4 ± 1.74	2.989 ± 0.69	0.453 ± 0.08	6.784 ± 0.31	0.774 ± 0.3	0.644 ± 0.08	2218.55 ± 26.8	8.782 ± 0.05	9.668 ± 0.29	0.045 ± 0.01	4.717 ± 0.38	1.024 ± 0.02	0.025 ± 0.02	1.324 ± 0.08	0.696 ± 0.03	0.039 ± 0.02	607.671 ± 18.39	81.435 ± 0.32	3.447 ± 0.21	62.61 ± 1.76	2.828 ± 0.04	39.099 ± 0.08	185.713 ± 2.23
M_B_21	23.25 ± 0.04	4.575 ± 0.01	n.d.	27.488 ± 1.4	0.130 ± 0.00	16.724 ± 0.71	377.3 ± 4.95	2.529 ± 0.04	0.331 ± 0.01	0.241 ± 0.02	4.651 ± 0.1	0.675 ± 0.02	1.572 ± 0.1	0.377 ± 0.01	2.298 ± 0.02	0.014 ± 0.02	574.831 ± 3.20	46.139 ± 0.85	5.453 ± 0.33	58.126 ± 0.14	0.403 ± 0.02	32.03 ± 0.35	229.809 ± 0.63
PN_B_19	109.36 ± 1.41	16.384 ± 1.41	0.078 ± 0.01	1.827 ± 0.03	0.478 ± 0.04	0.304 ± 0.02	1592.19 ± 7.07	3.281 ± 0.05	3.416 ± 0.18	0.029 ± 0.00	1.244 ± 0.01	0.802 ± 0.01	0.107 ± 0.04	1.588 ± 0.06	6.775 ± 0.19	0.44 ± 0.04	570.123 ± 3.68	76.945 ± 1.76	13.49 ± 0.42	61.734 ± 0.99	8.176 ± 0.41	35.197 ± 0.87	185.372 ± 1.20
PN_B_21	31.47 ± 0.49	20.250 ± 0.7	n.d.	33.136 ± 0.7	0.194 ± 0.01	20.737 ± 1.41	503.52 ± 2.12	2.995 ± 0.39	0.255 ± 0.01	0.322 ± 0.14	5.983 ± 0.48	1.002 ± 0.15	2.113 ± 0.32	0.41 ± 0.06	3.602 ± 0.41	0.037 ± 0.02	573.4975 ± 3.91	64.266 ± 1.14	3.302 ± 0.20	62.4 ± 0.99	n.d.	32.813 ± 1.90	204.074 ± 2.36
B_S_19	212.43 ± 1.42	42.457 ± 1.41	6.503 ± 0.00	23.083 ± 1.6	0.168 ± 0.00	2.114 ± 0.02	3129.48 ± 70.7	9.965 ± 0.34	4.448 ± 0.00	0.017 ± 0.00	3.744 ± 0.92	1.5 ± 0.02	0.251 ± 0.01	1.724 ± 0.01	n.d.	0.217 ± 0.00	593.233 ± 2.19	97.644 ± 1.01	37.065 ± 1.0	78.281 ± 2.25	14.105 ± 0.46	35.973 ± 1.35	106.129 ± 2.32
CF_S_19	26.17 ± 0.7	2.639 ± 0.17	5.532 ± 0.15	9.225 ± 0.71	0.556 ± 0.01	1.018 ± 0.00	302.00 ± 6.37	9.507 ± 0.05	6.45 ± 0.35	0.048 ± 0.00	4.676 ± 0.08	1.343 ± 0.03	0.095 ± 0.00	0.639 ± 0.2	0.487 ± 0.04	0.162 ± 0.03	614.405 ± 3.10	86.465 ± 0.84	16.796 ± 0.78	58.253 ± 0.63	1.561 ± 0.07	31.166 ± 0.34	67.09 ± 1.41
CS_D_19	3.86 ± 0.25	6.121 ± 0.21	1.028 ± 0.03	1.547 ± 0.03	0.115 ± 0.01	0.301 ± 0.02	55.79 ± 0.71	1.655 ± 0.13	0.485 ± 0.05	0.856 ± 0.21	2.336 ± 0.17	0.189 ± 0.06	0.088 ± 0.56	0.171 ± 0.38	1.168 ± 0.03	0.028 ± 0.00	549.574 ± 28.28	55.288 ± 2.83	11.76 ± 1.23	48.618 ± 1.02	1.15 ± 0.04	31.054 ± 0.36	100.365 ± 1.21
CS_D_21	69.58 ± 1.42	10.153 ± 0.46	2.271 ± 0.04	130.99 ± 0.7	0.451 ± 0.02	87.661 ± 1.42	761.77 ± 14.14	3.677 ± 0.05	3.272 ± 0.31	0.076 ± 0.01	10.351 ± 0.49	1.302 ± 0.01	0.025 ± 0.00	1.718 ± 0.04	18.904 ± 1.3	0.026 ± 0.03	584.299 ± 7.29	48.349 ± 0.34	7.543 ± 0.21	52.446 ± 1.31	7.146 ± 0.22	35.045 ± 1.13	147.668 ± 4.24
M_D_19	4.70 ± 0.01	7.274 ± 0.03	0.915 ± 0.02	1.06 ± 0.02	0.096 ± 0.01	0.246 ± 0.00	73.93 ± 0.46	2.335 ± 0.23	0.759 ± 0.03	0.576 ± 0.01	3.479 ± 0.22	0.166 ± 0.02	0.017 ± 0.05	0.16 ± 0.00	0.763 ± 0.02	0.024 ± 0.01	575.073 ± 3.07	79.33 ± 2.84	8.416 ± 0.20	61.788 ± 1.78	0.731 ± 0.02	33.643 ± 1.69	105.526 ± 3.78
ND_D_21	52.96 ± 1.42	14.110 ± 0.08	3.924 ± 0.49	94.71 ± 1.18	0.337 ± 0.00	50.825 ± 1.41	543.69 ± 0.71	6.756 ± 0.14	5.067 ± 0.43	n.d.	7.409 ± 0.05	0.948 ± 0.14	0.017 ± 0.11	1.9 ± 0.53	13.601 ± 0.5	0.025 ± 0.13	585.591 ± 3.86	68.718 ± 1.79	10.466 ± 0.05	53.587 ± 1.22	2.203 ± 0.13	31.993 ± 1.20	121.473 ± 2.25
ND_D_19	9.20 ± 0.12	12.805 ± 0.57	1.332 ± 0.02	4.634 ± 0.42	0.229 ± 0.01	0.576 ± 0.03	123.21 ± 1.57	4.766 ± 0.33	0.299 ± 0.04	0.198 ± 0.03	1.341 ± 0.13	0.232 ± 0.04	0.384 ± 0.04	0.312 ± 0.05	1.144 ± 0.02	0.052 ± 0.00	583.28 ± 4.58	73.061 ± 1.39	12.711 ± 1.84	66.51 ± 1.70	2.817 ± 0.14	34.667 ± 1.25	101.596 ± 0.45
M_F_19	26.49 ± 0.7	1.893 ± 0.36	0.596 ± 0.02	20.463 ± 0.4	0.043 ± 0.02	8.458 ± 0.5	204.37 ± 2.83	0.523 ± 0.21	0.082 ± 0.00	0.121 ± 0.01	2.234 ± 0.13	1.036 ± 0.00	3.008 ± 0.53	0.286 ± 0.02	0.407 ± 0.21	1.726 ± 0.33	55.966 ± 1.93	24.657 ± 1.74	184.174 ± 3.41	8.21 ± 0.54	0.955 ± 0.03	2.835 ± 0.35	13.091 ± 1.44
B_SF_19	139.80 ± 2.12	14.946 ± 1.06	0.792 ± 0.05	25.246 ± 1.4	0.178 ± 0.05	9.272 ± 0.39	1090.76 ± 16.3	15.543 ± 0.71	8.815 ± 0.02	0.026 ± 0.02	2.89 ± 0.13	0.675 ± 0.02	0.053 ± 0.03	0.508 ± 0.03	1.041 ± 0.05	0.874 ± 0.5	98.72 ± 1.95	26.41 ± 1.73	42.774 ± 1.67	21.396 ± 1.91	17.204 ± 0.58	5.57 ± 0.28	23.193 ± 1.60
B_SF_21	89.12 ± 0.01	15.439 ± 0.34	0.780 ± 0.01	23.66 ± 0.5	0.161 ± 0.01	5.109 ± 0.05	590.76 ± 9.17	18.275 ± 0.27	9.81 ± 0.51	0.007 ± 0.00	3.133 ± 0.01	0.485 ± 0.02	0.162 ± 0.00	0.86 ± 0.05	1.289 ± 0.00	12.301 ± 0.05	65.635 ± 1.19	710.951 ± 5.6	478.71 ± 4.43	22.395 ± 1.02	24.356 ± 1.24	4.805 ± 0.34	21.514 ± 0.97
CS_SF_19	6.13 ± 0.22	2.004 ± 0.26	0.507 ± 0.01	0.239 ± 0.01	0.044 ± 0.01	0.029 ± 0.02	57.34 ± 1.41	1.347 ± 0.11	0.403 ± 0.01	0.078 ± 0.11	1.371 ± 0.01	0.116 ± 0.02	0.016 ± 0.1	0.156 ± 0.00	0.385 ± 0.03	0.026 ± 0.07	61.53 ± 3.03	9.116 ± 0.37	6.015 ± 0.05	5.896 ± 0.05	0.845 ± 0.02	3.413 ± 0.06	16.89 ± 0.61
CS_SF_21	17.70 ± 0.28	3.937 ± 0.4	2.345 ± 0.29	0.687 ± 0.06	0.441 ± 0.02	0.168 ± 0.03	188.76 ± 2.13	17.036 ± 0.26	5.456 ± 0.04	0.025 ± 0.00	1.283 ± 0.01	0.689 ± 0.03	0.003 ± 0.00	0.733 ± 0.01	1.563 ± 0.15	0.156 ± 0.19	609.079 ± 5.87	116.329 ± 1.8	26.2950 ± 0.44	61.692 ± 0.56	8.198 ± 0.46	31.736 ± 0.98	69.662 ± 2.43
FN_SF_19	21.41 ± 0.52	1.733 ± 0.03	1.177 ± 0.05	2.937 ± 0.4	0.044 ± 0.01	0.519 ± 0.01	209.05 ± 2.13	0.95 ± 0.03	0.159 ± 0.01	0.212 ± 0.06	0.141 ± 0.01	0.191 ± 0.06	0.123 ± 0.05	0.503 ± 0.02	0.186 ± 0.02	0.039 ± 0.00	57.982 ± 1.94	13.273 ± 1.09	5.864 ± 0.16	6.472 ± 0.17	1.154 ± 0.27	4.124 ± 0.48	18.799 ± 1.44
FN_SF_21	146.40 ± 1.42	12.047 ± 0.18	1.931 ± 0.03	56.406 ± 1.4	0.143 ± 0.01	28.990 ± 1.42	1298.41 ± 28.3	57.026 ± 1.42	11.807 ± 0.71	0.088 ± 0.04	7.245 ± 0.52	1.362 ± 0.18	0.098 ± 0.04	2.021 ± 0.13	1.527 ± 0.19	0.378 ± 0.3	82.083 ± 0.89	26.337 ± 1.84	50.582 ± 1.25	24.619 ± 0.66	38.215 ± 0.49	5.481 ± 0.23	27.812 ± 1.91
M_SF_21	30.89 ± 0.66	24.523 ± 0.5	0.605 ± 0.03	1.032 ± 0.2	0.197 ± 0.02	0.431 ± 0.09	444.40 ± 14.84	2.266 ± 0.05	0.236 ± 0.02	n.d.	3.001 ± 0.09	0.454 ± 0.06	0.017 ± 0.00	0.17 ± 0.01	1.65 ± 0.4	0.666 ± 0.03	540.442 ± 5.11	86.785 ± 1.64	49.874 ± 3.14	55.454 ± 1.67	1.858 ± 0.20	41.791 ± 1.23	96.025 ± 4.08
PN_SF_19	19.72 ± 0.4	2.725 ± 0.5	0.140 ± 0.2	10.601 ± 1.1	0.032 ± 0.2	2.753 ± 0.38	199.87 ± 2.83	0.955 ± 0.03	0.274 ± 0.03	0.288 ± 0.03	1.55 ± 0.32	1.4 ± 0.02	2.278 ± 0.05	0.363 ± 0.02	0.083 ± 0.04	4.873 ± 0.45	57.432 ± 1.33	574.68 ± 14.1	294.661 ± 3.93	11.042 ± 0.53	3.79 ± 0.12	7.28 ± 0.21	25.497 ± 1.41
PN_SF_21	168.47 ± 1.41	26.885 ± 0.39	1.979 ± 0.00	61.668 ± 0.4	0.146 ± 0.00	29.778 ± 1.17	1504.92 ± 21.2	28.042 ± 0.07	4.194 ± 0.53	6.634 ± 0.21	12.38 ± 0.41	0.96 ± 0.09	0.225 ± 0.04	1.746 ± 0.2	0.449 ± 0.01	0.973 ± 0.18	82.227 ± 1.09	30.847 ± 0.83	57.778 ± 2.81	44.986 ± 1.54	48.136 ± 1.81	5.577 ± 0.13	23.233 ± 1.43
CD_BN_19	9.73 ± 0.01	1.434 ± 0.0	0.178 ± 0.0	7.604 ± 0.0	0.040 ± 0.0	3.699 ± 0.0	293.71 ± 0.01	1.938 ± 0.00	0.24 ± 0.00	0.198 ± 0.0	0.605 ± 0.00	2.369 ± 0.00	5.493 ± 0.00	0.276 ± 0.00	0.02 ± 0.00	0.629 ± 0.00	18.466 ± 0.89	n.d.	81.288 ± 1.10	7.382 ± 0.31	3.701 ± 0.16	5.321 ± 0.08	7.733 ± 0.28
FN_C_19	13.08 ± 0.46	1.170 ± 0.01	0.201 ± 0.00	3.731 ± 0.29	0.047 ± 0.00	1.443 ± 0.01	548.45 ± 21.12	3.435 ± 0.06	0.359 ± 0.02	0.46 ± 0.04	1.999 ± 0.03	14.472 ± 0.53	4.499 ± 0.35	0.161 ± 0.01	0.118 ± 0.01	1.357 ± 0.03	107.469 ± 4.95	48.115 ± 1.57	136.634 ± 2.23	13.218 ± 1.02	1.188 ± 0.46	39.973 ± 2.58	9.975 ± 0.64
PN_T_19	6.96 ± 0.00	1.220 ± 0.00	0.164 ± 0.00	20.484 ± 0.7	0.007 ± 0.0	7.441 ± 0.21	199.94 ± 2.83	0.83 ± 0.00	0.317 ± 0.0	0.192 ± 0.0	2.967 ± 0.01	1.23 ± 0.01	1.357 ± 0.0	0.158 ± 0.0	n.d.	0.054 ± 0.0	24.56 ± 2.51	n.d.	16.231 ± 0.79	9.611 ± 0.35	n.d.	4.454 ± 0.31	6.422 ± 0.18

n.d.—not determined; 3,4,DHBA—3,4 dihydroxybenzoic acid; 4,HBA—4,hydroxybenzoic acid.

## Data Availability

All data related to the manuscript are available in the manuscript and the Supplementary Materials.

## Supplementary Materials

The following supporting information can be downloaded at: https://www.mdpi.com/article/10.3390/antiox14050564/s1, Table S1. The identification of phenolic compounds in grape must and wine by UHPLC–ESI/HRMS with structures confirmed by comparison with reference standards, Table S2. References data, Table S3. Correlation matrix and Pearson coefficients of determination for individual phenolic compounds in wine for white grape cultivars, Table S4. Correlation matrix and Pearson coefficients of determination for individual phenolic compounds in wine for red grape cultivars.
