# Supplementary material for: Phytochemical Screening and Antioxidant Activities of White and Red Wines from Different Varieties and Wine Regions in Romania
Source: Antioxidants (Basel). 2025 May 8;14(5):564. doi: 10.3390/antiox14050564 (PMC12108437; doi:10.3390/antiox14050564)
Supplement: Supplementary file 1 [file antioxidants-14-00564-s001.zip › antioxidants-3580102-supplementary.pdf]

SUPPLEMENTARY INFORMATION

Table S1. The identification of phenolic compounds in grape must and wine by UHPLC–ESI/HRMS with structures confirmed by comparison with reference standards.

No	Compound	Retention Time (min)	Formula	Exact Mass	Accurate mass (M-H)⁻	Experimental Adduct Ion (m/z)	Mass Fragments
	**Phenolic acids**						
1	Gallic acid	1.94	$C_7H_6O_5$	170.0215	169.0142	169.0133	125.0231
2	3,4-Dihydroxybenzoic acid	4.25	$C_7H_6O_4$	154.0266	153.0193	153.0184	109.0281
3	4-Hydroxybenzoic acid	6.96	$C_7H_6O_3$	138.0316	137.0243	137.0233	118.9650, 96.9588, 71.0124
4	t-Ferulic acid	8.89	$C_{10}H_{10}O_4$	194.0579	193.0506	193.0499	178.0262, 134.0361
5	Chlorogenic acid	7.90	$C_{16}H_{18}O_9$	354.0950	353.0877	353.0880	191.0553
7	Syringic acid	8.44	$C_9H_{10}O_5$	198.0528	197.0455	197.0450	182.0212, 166.9976, 153.0547, 138.0311, 123.0075
8	Cinnamic acid	8.45	$C_9H_8O_2$	148.0524	147.0451	147.0442	119.0489, 103.0387
9	Ellagic acid	9.69	$C_{14}H_6O_8$	302.0062	300.9989	300.9993	300.9990
10	p-Coumaric acid	8.72	$C_9H_8O_3$	164.0473	163.0400	163.0390	119.0489
	**Flavonoids**						
	Catechin	7.57	$C_{15}H_{14}O_6$	290.0790	289.0717	289.0719	109.0282, 123.0349, 125.0232, 137.0232, 151.0390, 203.0708
	Epi-catechin	8.14	$C_{15}H_{14}O_6$	290.0790	289.0717		
	Quercetin	10.66	$C_{15}H_{10}O_7$	302.2357	301.0354	301.0356	151.0226, 178.9977, 121.0282, 107.0125
	Isorhamnetin	11.79	$C_{16}H_{12}O_7$	316.0582	315.0509	315.0515	300.0277
	Kaempferol	11.59	$C_{15}H_{10}O_6$	286.0477	285.0404	285.0406	151.0389, 117.0180
	Apigenin	11.86	$C_{15}H_{10}O_5$	270.0528	269.0455	269.0455	117.0333, 151.0027, 107.0126
	Pinocembrin	12.58	$C_{15}H_{12}O_4$	256.0735	255.0662	255.0663	213.0551, 151.0026, 107.0125
	Chrysin	13.41	$C_{15}H_{10}O_4$	254.0579	253.0506	253.0505	143.0491, 145.0284, 107.0125, 209.0603, 63.0226, 65.0019
	Galangin	13.68	$C_{15}H_{10}O_5$	270.0528	269.0455	269.0455	169.0650, 143.0491
	Naringin	11.9	$C_{27}H_{32}O_{14}$	580.1792	217.33	258.5	177, 151
	**Stilbens**						
	t-Resveratrol	9.55	$C_{14}H_{12}O_3$	228.0786	227.0713	227.0707	185.0813, 143.0337

Table S2. Content of total phenolic compounds (TPC, expressed as mg GAE/L), total anthocyanins compounds (TA, expressed in mg/L), TCC – total catechins compounds catechine/ flavonoid mg/L, TTC- total tannins compounds (expressed in mg/L), AA-antioxidant activity (expressed in mgGAE/L or mMol/L), CI (color intensity), H (Hue) in wine.

Wine	TPC (mg GAE/L)	AA	TA (mg/L)	TCC (mg/L)	TTC (mg/L)	CI	H	references
Madeira	469.98+13.63<br>332.17+9.58<br>474.15+15.64<br>444.01+17.21	-	-	-	-	-	-	Pereira et. al., 2013
Shiraz wine	2064	13.01 ± 2.24<br>µmol TE equiv	198±93	17.8±9.0	1293±203	-	-	Irine R. Ginjom, (2010)
Cabernet Sauvignon wine	2382	15.9± 2.27<br>µmol TE equiv	190±54	10.8±5.32	1476±395	-	-	
Merlot wine	2518	15.21± 3.12<br>µmol TE equiv	134±38.4	7.98±10.02	1312±333	-	-	
Chardonnay wine	445	1.54± 0.1<br>µmol TE equiv	--	-	-	-	-	
Cabernet Sauvignon	2081-2486l	19.2 mMol/L	-	-	-	-	-	Landrault et al., (2001)
Syrah	2293-2338	20.2 mMol/L	-	-	-	-	-	
Merlot	2200-2239	19.9 mMol/L	-	-	-	-	-	
Pinot Noir	2329	21.2 mMol/L	-	-	-	-	-	
Sauvignon 1998	262	1.69 mMol/L	-	-	-	-	-	
Chardonnay1999	379	2.31 mMol/L	-	-	-	-	-	
Terret Sauvignon1998	289	2.11 mMol/L	-	-	-	-	-	
Merlot	2091 ± 57	0.87 ± 0.01 mMol/L	143.5 ± 0.7	442.9 ± 3.8	-	-	-	Anis Arnous, (2002)
Syrah	2439 ± 278	1.14 ± 0.05 mMol/L	332.4 ± 5.9	650.4 ± 19.6	-	-	-	
Cabernet Sauvignon	3772 ± 284	1.37 ± 0.05 mMol/L	360.1 ± 9.9	664.8 ± 9.5	-	-	-	
Merlot	1,714	-	387	2170	2721	-	-	
Cabernet Sauvignon	1329 ± 36	0.07 ± 0.00<br>DPPH - IC50<br>(mg/mL)	-	-	-	-	-	Beara,(2018)
Riesling Italian	2162 ± 23	0.09 ± 0.01<br>DPPH - IC50<br>(mg/mL)	-	-	-	-	-	

Feteasca Regala 2012 – Recaș	244	0.82 (mMol/L)	-	-	-	-	-	Banc R et al., (2020)
Feteasca Neaga, 2011 Murfatlar 2012 Ceptura	1660/2248.00	5.54 / 8.69 (mMol/L)	-	-	-	-	-	
Babeasca Rose, 2012 Panciu	236	0,86 (mMol/L)	-	-	-	-	-	
Merlot	1720	17,6 mMol/L	-	-	-	-	-	Anli and Vural, 2009
Cabernet Sauvignon	2320	18,1 mMol/L	-	-	-	-	-	
Chardonnay different regions	403.64 ± 9.41 l<br>329.89 ± 9.41<br>339.72 ± 9.41<br>396.26 ± 23.41	59.43 ± 0.30 mMol/L<br>60.32 ± 1.03 mMol/L<br>61.60 ± 0.24 mMol/L<br>71.87 ± 0.95 mMol/L	-	-	-	-	-	Violeta Ivanova-Petropulos, 2021
Pinot Gris different regions	307.77 ± 20.47<br>268.44 ± 8.03<br>256.15 ± 9.41<br>381.51 ± 8.03	67.40 ± 0.44 mMol/L<br>72.53 ± 0.35 mMol/L<br>51.06 ± 0.27 mMol/L<br>66.80 ± 0.66 mMol/L	-	-	-	-	-	
Cabernet Sauvignon	3377.6 ± 369.6	-	681.8 ± 100.8	2.7 × 10<br>−3 ± 1.7 × 10<br>−4 mol cat/l	79.1 ± 1.7	15.2 ± 0.9	10.8 ± 1.9	Fanzonea et al., 2012
Merlot	3447.5 ± 372.3 l	-	644.1 ± 37.6	2.2 × 10<br>−3 ± 2.0 × 10<br>−4 mol cat/l	79.2 ± 4.2	17.3 ± 0.5	19.2 ± 1.9	
Shiraz	1585.6 ± 50.6	-	301.4 ± 18.9	8.8 × 10<br>−4 ± 3.7 × 10<br>−5 mol cat/l	75.1 ± 1.7	5.8 ± 0.1	3.6 ± 2.0	
Cabernet Sauvignon	3087	82.2 mg GAE/L	305	12.53	2813.2 mg GAE/L	-	-	Katalinic et al., 2004
Merlot	2402	68.86 mg GAE/L	146.2	7.29	2105.2 mg GAE/L	-	-	

[1] Pereira et. al., 2013; [2] Irine R. Ginjom, 2010; [3] Landrault et al., 2001; [4] Anis Arnous, 2002; [5] Beara,2018; [6] Banc R et al.,2020; [7] Anli and Vural, 2009; [8] Ivanova-Petropulos, 2021; [9] Fanzonea et al., 2012; [10] Katalinic et al., 2004.

Table S3. Correlation matrix and Pearson coefficients of determination for individual phenolic compounds in wine for white grape cultivars.

Variables	galic acid	3,4 DHB	4 HBA	catechin	epi-catechi	siringic acid	p-coumari	ferulic acid	resveratrol	elagic acid	abscisic acid	cinamic aci	quercitin
galic acid	**1**	**0.808**	**0.551**	**0.289**	0.093	**0.968**	**0.615**	**0.630**	**0.689**	**0.695**	**0.702**	-0.003	**0.299**
3,4 DHBA	**0.808**	**1**	**0.616**	**0.296**	0.158	**0.711**	**0.455**	**0.599**	**0.676**	**0.559**	**0.635**	0.195	**0.398**
4 HBA	**0.551**	**0.616**	**1**	**0.393**	**0.360**	**0.469**	**0.682**	**0.651**	**0.766**	**0.798**	**0.772**	**0.232**	**0.602**
catechin	**0.289**	**0.296**	**0.393**	**1**	**0.877**	**0.227**	**0.301**	**0.325**	**0.530**	**0.371**	**0.415**	0.118	**0.260**
epi-catechin	0.093	0.158	**0.360**	**0.877**	**1**	0.069	0.196	0.090	**0.410**	**0.224**	**0.304**	0.170	0.213
siringic acid	**0.968**	**0.711**	**0.469**	**0.227**	0.069	**1**	**0.561**	**0.557**	**0.600**	**0.629**	**0.651**	-0.033	0.200
p-coumaric acid	**0.615**	**0.455**	**0.682**	**0.301**	0.196	**0.561**	**1**	**0.823**	**0.713**	**0.921**	**0.906**	**0.260**	0.122
ferulic acid	**0.630**	**0.599**	**0.651**	**0.325**	0.090	**0.557**	**0.823**	**1**	**0.701**	**0.844**	**0.858**	**0.398**	0.114
resveratrol	**0.689**	**0.676**	**0.766**	**0.530**	**0.410**	**0.600**	**0.713**	**0.701**	**1**	**0.821**	**0.802**	**0.431**	**0.435**
elagic acid	**0.695**	**0.559**	**0.798**	**0.371**	**0.224**	**0.629**	**0.921**	**0.844**	**0.821**	**1**	**0.935**	**0.256**	**0.252**
abscisic acid	**0.702**	**0.635**	**0.772**	**0.415**	**0.304**	**0.651**	**0.906**	**0.858**	**0.802**	**0.935**	**1**	**0.276**	0.198
cinamic acid	-0.003	0.195	**0.232**	0.118	0.170	-0.033	**0.260**	**0.398**	**0.431**	**0.256**	**0.276**	**1**	-0.029
quercitin	**0.299**	**0.398**	**0.602**	**0.260**	0.213	0.200	0.122	0.114	**0.435**	**0.252**	0.198	-0.029	**1**

Values in bold are different from 0 with a significance level alpha=0.05.

Table S4. Correlation matrix and Pearson coefficients of determination for individual phenolic compounds in wine for red grape cultivars.

Variables	galic acid	3,4 DHBA	4,HBA	catechin	clorogenic acid	epi-catechin	siringic acid	p-cumaric acid	ferulic acid	naringin	resveratrol	elagic acid	myrcetin	abscisic acid	cinamic acid	quercitin	kaemferol	isorhamnetin	apigenin	pinocembrin	CAPE	crysin	galangin
galic acid	**1**	**0.731**	**0.438**	**0.484**	0.263	0.364	**0.916**	**0.698**	**0.731**	**0.427**	**0.632**	-0.165	-0.374	**0.774**	0.099	0.131	0.054	0.062	0.258	-0.038	**0.823**	-0.154	0.072
3,4 DHBA	**0.731**	**1**	**0.470**	0.329	0.234	0.239	**0.717**	0.365	0.360	0.404	**0.536**	-0.252	**-0.447**	**0.537**	0.149	0.106	0.091	0.009	**0.582**	0.320	**0.564**	0.209	0.341
4,HBA	**0.438**	**0.470**	**1**	0.387	**0.549**	0.326	**0.485**	0.308	0.395	0.097	0.401	-0.195	**-0.430**	**0.619**	0.329	-0.174	-0.088	-0.239	**0.588**	**0.509**	0.251	0.278	0.317
catechin	**0.484**	0.329	0.387	**1**	0.303	**0.980**	0.378	0.380	0.382	0.276	**0.829**	-0.134	-0.242	**0.768**	**0.797**	-0.033	-0.062	-0.079	0.193	0.098	**0.448**	-0.038	0.254
clorogenic acid	0.263	0.234	**0.549**	0.303	**1**	0.332	0.272	0.189	**0.459**	-0.066	0.305	-0.209	**-0.456**	**0.464**	**0.513**	-0.104	0.006	-0.208	**0.719**	**0.733**	0.086	**0.514**	**0.645**
epi-catechin	0.364	0.239	0.326	**0.980**	0.332	**1**	0.277	0.296	0.281	0.219	**0.781**	-0.102	-0.208	**0.697**	**0.860**	-0.118	-0.128	-0.155	0.205	0.161	0.339	0.045	0.316
siringic acid	**0.916**	**0.717**	**0.485**	0.378	0.272	0.277	**1**	**0.518**	**0.540**	0.334	**0.524**	0.029	-0.206	**0.704**	0.083	-0.011	-0.055	-0.043	0.328	0.075	**0.619**	0.071	0.179
p-cumaric aci	**0.698**	0.365	0.308	0.380	0.189	0.296	**0.518**	**1**	**0.814**	0.287	**0.535**	-0.103	-0.319	**0.712**	-0.022	0.106	0.038	0.060	0.128	-0.113	**0.860**	-0.217	-0.115
ferulic acid	**0.731**	0.360	0.395	0.382	**0.459**	0.281	**0.540**	**0.814**	**1**	0.015	0.401	-0.192	**-0.443**	**0.666**	0.143	0.341	0.262	0.225	0.196	0.025	**0.691**	-0.152	0.023
naringin	**0.427**	0.404	0.097	0.276	-0.066	0.219	0.334	0.287	0.015	**1**	**0.661**	-0.011	-0.084	0.353	-0.108	-0.049	-0.109	-0.057	0.133	-0.157	**0.650**	-0.163	-0.126
resveratrol	**0.632**	**0.536**	0.401	**0.829**	0.305	**0.781**	**0.524**	**0.535**	0.401	**0.661**	**1**	-0.114	-0.339	**0.768**	**0.481**	-0.071	-0.107	-0.130	0.363	0.134	**0.690**	0.027	0.247
elagic acid	-0.165	-0.252	-0.195	-0.134	-0.209	-0.102	0.029	-0.103	-0.192	-0.011	-0.114	**1**	**0.639**	-0.192	-0.109	-0.006	-0.097	0.141	-0.254	-0.201	-0.148	0.368	-0.253
myrcetin	-0.374	**-0.447**	**-0.430**	-0.242	**-0.456**	-0.208	-0.206	-0.319	**-0.443**	-0.084	-0.339	**0.639**	**1**	-0.400	-0.253	0.018	-0.074	0.227	**-0.508**	**-0.430**	-0.297	-0.054	-0.389
abscisic acid	**0.774**	**0.537**	**0.619**	**0.768**	**0.464**	**0.697**	**0.704**	**0.712**	**0.666**	0.353	**0.768**	-0.192	-0.400	**1**	**0.508**	0.013	0.016	-0.055	0.375	0.174	**0.716**	-0.011	0.266
cinamic acid	0.099	0.149	0.329	**0.797**	**0.513**	**0.860**	0.083	-0.022	0.143	-0.108	**0.481**	-0.109	-0.253	**0.508**	**1**	-0.106	-0.051	-0.170	0.354	**0.451**	-0.036	0.330	**0.553**
quercitin	0.131	0.106	-0.174	-0.033	-0.104	-0.118	-0.011	0.106	0.341	-0.049	-0.071	-0.006	0.018	0.013	-0.106	**1**	**0.915**	**0.958**	-0.209	-0.294	0.256	-0.260	-0.237
kaemferol	0.054	0.091	-0.088	-0.062	0.006	-0.128	-0.055	0.038	0.262	-0.109	-0.107	-0.097	-0.074	0.016	-0.051	**0.915**	**1**	**0.889**	-0.055	-0.105	0.144	-0.131	-0.069
isorhamnetin	0.062	0.009	-0.239	-0.079	-0.208	-0.155	-0.043	0.060	0.225	-0.057	-0.130	0.141	0.227	-0.055	-0.170	**0.958**	**0.889**	**1**	-0.314	-0.378	0.194	-0.258	-0.331
apigenin	0.258	**0.582**	**0.588**	0.193	**0.719**	0.205	0.328	0.128	0.196	0.133	0.363	-0.254	**-0.508**	0.375	0.354	-0.209	-0.055	-0.314	**1**	**0.923**	0.110	**0.714**	**0.819**
pinocembrin	-0.038	0.320	**0.509**	0.098	**0.733**	0.161	0.075	-0.113	0.025	-0.157	0.134	-0.201	**-0.430**	0.174	**0.451**	-0.294	-0.105	-0.378	**0.923**	**1**	-0.230	**0.817**	**0.878**
CAPE	**0.823**	**0.564**	0.251	**0.448**	0.086	0.339	**0.619**	**0.860**	**0.691**	**0.650**	**0.690**	-0.148	-0.297	**0.716**	-0.036	0.256	0.144	0.194	0.110	-0.230	**1**	-0.331	-0.176
crysin	-0.154	0.209	0.278	-0.038	**0.514**	0.045	0.071	-0.217	-0.152	-0.163	0.027	0.368	-0.054	-0.011	0.330	-0.260	-0.131	-0.258	**0.714**	**0.817**	-0.331	**1**	**0.686**
galangin	0.072	0.341	0.317	0.254	**0.645**	0.316	0.179	-0.115	0.023	-0.126	0.247	-0.253	-0.389	0.266	**0.553**	-0.237	-0.069	-0.331	**0.819**	**0.878**	-0.176	**0.686**	**1**

Values in bold are different from 0 with a significance level alpha=0.05.
